# *De novo* biosynthesis of myricetin, kaempferol and quercetin in *Streptomyces albus* and *Streptomyces coelicolor*

**DOI:** 10.1371/journal.pone.0207278

**Published:** 2018-11-15

**Authors:** Laura Marín, Ignacio Gutiérrez-del-Río, Rodrigo Entrialgo-Cadierno, Claudio J. Villar, Felipe Lombó

**Affiliations:** 1 Research Group BIONUC (Biotechnology of Nutraceuticals and Bioactive Compounds), Departamento de Biología Funcional, Área de Microbiología, Universidad de Oviedo, Oviedo, Principality of Asturias, Spain; 2 IUOPA (Instituto Universitario de Oncología del Principado de Asturias) Principality of Asturias, Spain; 3 ISPA (Instituto de Investigación Sanitaria del Principado de Asturias), Principality of Asturias, Spain; Universite Paris-Sud, FRANCE

## Abstract

Flavonols are a flavonoid subfamily widely distributed in plants, including several ones of great importance in human and animal diet (apple, tomato, broccoli, onion, beans, tea). These polyphenolic nutraceuticals exert potent antimicrobial (membrane potential disruptors), antioxidant (free-radical scavengers), pharmacokinetic (CYP450 modulators), anti-inflammatory (lipoxygenase inhibitors), antiangiogenic (VEGF inhibitors) and antitumor (cyclin inhibitors) activities. Biotechnological production of these nutraceuticals, for example via heterologous biosynthesis in industrial actinomycetes, is favored since in plants these polyphenols appear as inactive glycosylated derivatives, in low concentrations or as part of complex mixtures with other polyphenolic compounds. In this work, we describe the *de novo* biosynthesis of three important flavonols, myricetin, kaempferol and quercetin, in the industrially relevant actinomycetes *Streptomyces coelicolor* and *S*. *albus*. *De novo* biosynthesis of kaempferol, myricetin and quercetin in actinomycetes has not been described before.

## Introduction

**Fig 1:**
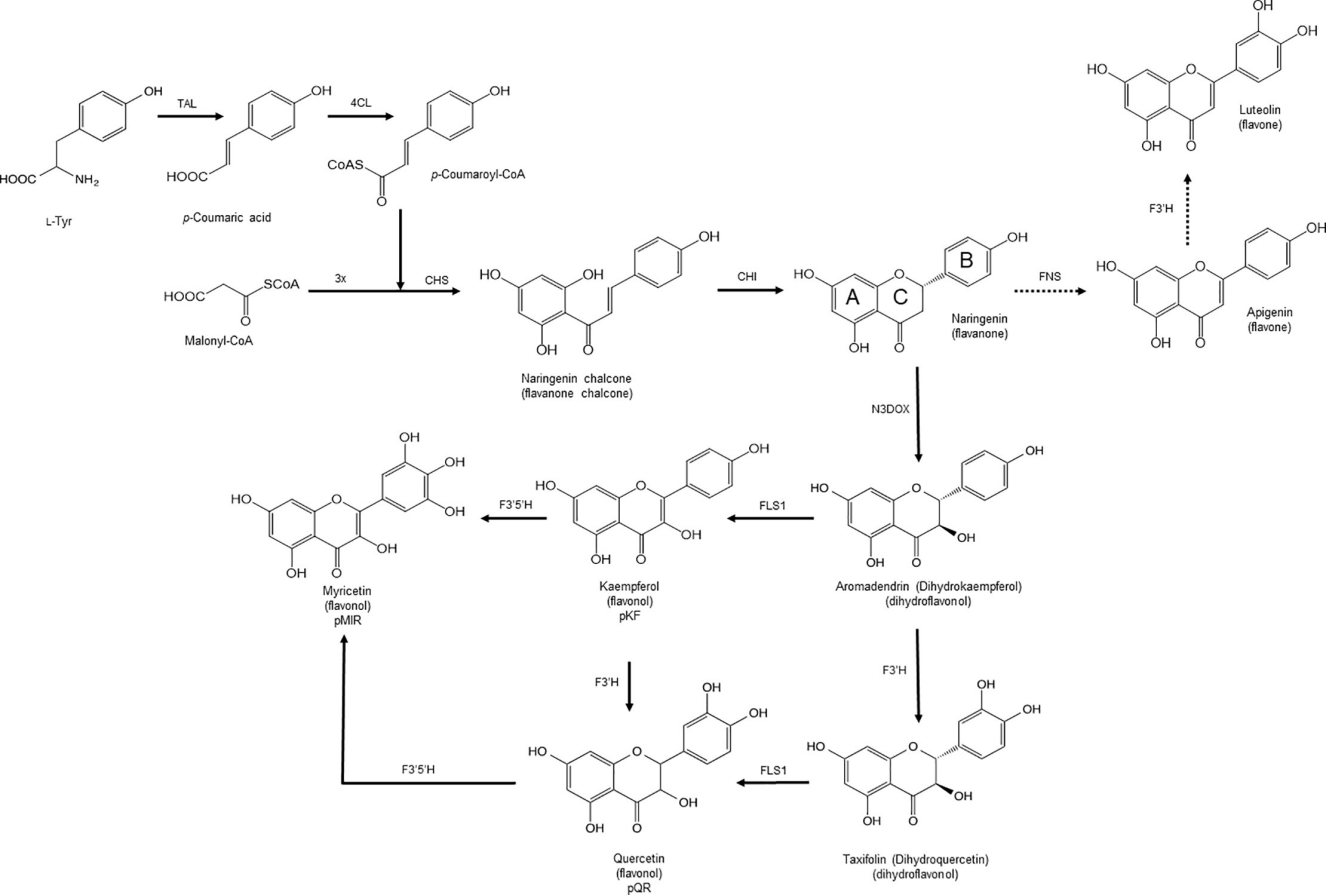
Engineered flavonoid biosynthetic pathway in *Streptomyces* sp., including the different feeding experiments with naringenin (dashed lines). Enzyme abbreviations: TAL, Tyrosine ammonia-lyase; 4CL, 4-coumaroyl CoA ligase; CHS, chalcone synthase; CHI, chalcone isomerase; N3DOX, naringenin 3-dioxygenase; FLS1, flavonol synthase 1; F3’H, flavonoid 3’-hydroxylase; F3’5’H, flavonoid 3’,5’-hydroxylase.

Flavonoids (from Latin *flavus*, yellow) are a family of about 6000 nutraceuticals widely distributed in plant cells, many of them found in dietary plants [1–5]. All flavonoids have a generic chemical structure consisting of 15 carbon atoms (C6-C3-C6): two aromatic rings (rings A and B) connected by a heterocyclic pyran C which contains one oxygen (ring C, Fig 1) [6–11]. This basic skeleton can have multiple substituents, such as hydroxyl or methyl groups, as well as sugars [12]; indeed, chemical modifications in ring C lead to the formation of more than 9,000 flavonoid derivates [13].

Depending on the pattern of hydroxylation and the substituents on the heterocyclic ring C, flavonoids can be classified into several sub-groups, but in this paper we will focus on flavonols. They are an important subfamily, as some of their members, like myricetin, kaempferol and quercetin, represent the major intake of dietary flavonoids in most societies [2,3,9]. Actually, quercetin is the most common flavonoid in human diet, with an average intake of 13 mg/day from a total of 20 to 50 mg/day for all flavonoids [14]. The reason for this is that quercetin concentration is very high in some vegetables, like in onions (1.2 g/kg) and cabbage (0.6 mg/kg), but also in many other fruits such as blueberries, apples, tomatoes or peaches [2]. Kaempferol is also present in many plant products: flowers (1.2 mg/kg in bee pollen, 205 mg/kg in saffron), fruits (29 mg/kg in beans) and vegetables (13 mg/kg in broccoli, 22 mg/kg in cabbage and 131 mg/kg in cappers) [15–18]. Finally, myricetin can be found in diverse food sources such as tea (940 mg/kg), grapes (15 mg/kg), blackcurrants (71 mg/kg), cranberries (142 mg/kg) and blueberries (26 mg/kg) [19–21]. As other flavonoids, flavonols commonly appear in these foods as glucose or rhamnose conjugates [22,23].

As antioxidant compounds, myricetin and quercetin are able to induce glutathione-S-transferase, an important enzyme involved in oxidative stress resistance [24]. Also, these flavonols are able to act directly as free-radical scavengers preventing DNA, protein and membrane damages thanks to their aromatic hydroxyl groups [25].

As well as other flavonoids, flavonols possess anti-inflammatory activities. For example, kaempferol and quercetin can inhibit tyrosine kinases involved in activated macrophage proliferation [26], and myricetin and quercetin are able to inhibit lipoxygenases, which catalyze important steps during formation of pro-inflammatory leukotrienes and hepoxilins [27]. Linked to these anti-inflammatory activities, kaempferol and quercetin are able to protect also against diverse pro-inflammatory and pro-carcinogenic agents, as they can bind to the aryl hydrocarbon receptor AhR (the activator of CYP1A1 and CYP1A2 transcription), therefore protecting cells against more reactive metabolites (on DNA, etc.) generated by the action of the detoxifying cytochromes P_450_ on polycyclic aromatics and halogenated toxins [28,29]. These effects, together with other ones, associated to cyclins inhibition and p53 concentrations increase carried out by myricetin and quercetin, contribute to the antitumor activity shown by flavonol nutraceuticals [30]. Myricetin, kaempferol and quercetin are also able to block angiogenesis, by inhibiting VEGF, another activity linked to antitumor properties [31].

Apart from these bioactivities in eukaryotic cells, flavonols are also important antimicrobial agents in the producer plants, as they are able to modify membrane transport mechanisms, altering membrane potential and leading to bacterial death [32,33].

*In planta*, flavonoids are synthesized by complexes of various enzymes that are present on the cytosolic face of endoplasmic reticulum membranes. The first steps for flavonoid biosynthesis are included in the phenylpropanoid pathway, which converts L-Phe in 4-coumaroyl-CoA in three steps [13,34]. These first three steps are catalyzed by phenylalanine ammonia lyase (PAL), cinnamate 4-hydroxylase (4CH) and 4-coumaroyl CoA ligase (4CL) (Fig 1). However, in bacteria, the use of tyrosine ammonia lyase (TAL) is preferred for heterologous biosynthesis, as starting from L-Tyr, the need for the 4CH activity (a plant membrane-bound enzyme) does not longer exist, as this amino acid is already hydroxylated at the required position [35,36]. Then, the chalcone synthase (CHS) condenses a molecule of 4-coumaroyl-CoA with three molecules of malonyl-CoA, generating naringenin chalcone, the basic skeleton for more than 9,000 flavonoids [3,13,34,37]. The heterocycle C closure is catalyzed by chalcone isomerase (CHI), which generates naringenin, the flavanone common precursor for all flavonols.

In order to generate kaempferol from naringenin, the action of naringenin 3-dioxygenase (N3DOX) is required to produce dihydrokaempferol (aromadendrin) and then the flavonol synthase 1 (FLS1) transforms this intermediate in kaempferol (Fig 1). Kaempferol is then the substrate for the flavonoid 3',5’-hydroxylase (F3'5’H), giving rise to myricetin (Fig 1). On the other hand, dihydrokaempferol is also the substrate for flavonoid 3’-hydroxylase (F3'H), generating dihydroquercetin (taxifolin) (Fig 1), which finally is transformed in quercetin by the action of flavonol synthase 1 (FLS1) [12].

Myricetin, kaempferol, quercetin and their dihydro precursors show interesting nutraceutical activities, as it has been described above. This makes these bioactive compounds attractive targets for genetic and metabolic engineering experiments, like the heterologous expression of their plant biosynthetic gene pathways in suitable microbial factories such as actinomycetes. In this work, we have carried out this by using combinatorial biosynthesis, where genes from different organisms are grouped in an artificial gene cluster directing the production of the natural bioactive compound [34,38]. Previous studies have reported the biosynthesis of flavonols in microorganisms; for instance, myricetin has been already heterologously produced, after feeding with naringenin precursor, in a strain of *E*. *coli* containing an incomplete flavonol biosynthetic gene cluster [39]. Also, kaempferol has been already produced in *E*. *coli* [40] and *Saccharomyces cerevisiae* [41], needing in some cases feeding with coumaric acid or naringenin [39]. In the case of its heterologous production in *Streptomyces venezuelae*, feeding with naringenin was also necessary [42]. Finally, quercetin has been also produced in *E*. *coli* and in *S*. *cerevisiae* after feeding with coumaric acid or naringenin [39,41].

In this work, we have achieved *de novo* production of these three flavonols by means of biosynthetic pathways heterologously expressed in *S*. *albus* and *S*. *coelicolor*, without feeding with precursors.

## Material and methods

### Bacterial strains, plasmids and culture conditions

*E*. *coli* TOP10 (Invitrogen) and pUC57 (Fermentas) were used for routine sub-cloning while *E*. *coli* ET12567 [43] was required to obtain non-methylated DNA for later protoplasts transformation in *Streptomyces coelicolor* M1154 [44]. The high-copy number *E*. *coli*-*Streptomyces* shuttle vector pIAGO, a derivative of pWHM3 which contains the strong constitutive promoter for ermE* (P_ermE*_) [45], was used as expression plasmid. The strain *Streptomyces albus* J1074 [46] was also used for the production of flavonols.

*E*. *coli* strains were grown in TSB liquid broth or TSB agar, supplemented with the corresponding antibiotics (ampicillin 100 μg/ml, Sigma Aldrich) for plasmid selection. *S*. *coelicolor* M1154 and *S*. *albus* J1074 were grown at 30°C in YEME 34% and 17% sucrose respectively, for protoplasts preparation. Both species were sporulated on SFM and Bennet medium respectively [47], supplemented with the corresponding antibiotics when necessary (thiostrepton 50 μg/mL).

For flavonols production, *S*. *albus* and *S*. *coelicolor* clones were grown on 3 ml of solid R5A medium [48], supplemented with the corresponding antibiotic, during 5 days at 30°C. Spores were previously quantified and an inoculum of 10^7^ spores/mL was used for each culture.

### DNA manipulation

Restriction enzymes were purchased from Takara Biochemicals, T4 DNA ligase from Thermo Scientific, and Dream Taq DNA Polymerase from Thermo Scientific. Synthetic genes for the following ORFs were generated by Genscript after codon optimization: *TAL* from *Rhodobacter capsulatus* (accession number WP_013066811), *4CL* from *S*. *coelicolor* (accession number NP_628552), *CHS* from *Glycine max* (accession number L07647.1), *CHI* from *G*. *max* (accession number AY595413.1), *N3DOX* from *Petroselinum crispum* (accession number AY23248), *FLS1* from *Arabidopsis thaliana* (accession number Q96330), *F3’H* from *A*. *thaliana* (accession number Q9SD85) and *F3’5’H* from *Petunia x hybrida* (accession number Z22544.1). Genbank accession numbers LT629805.1, LT629806.1, LT629807.1, LT629808.1, LT629809.1, MG748610, MG748611 and MG748612 for synthetic genes TAL, 4CL, CHS, CHI, F3’H, N3DOX, FLS1 and F3’5’H respectively. Compatible restriction sites were added at each gene cassette end, in order to facilitate construction of the recombinant flavonoids gene clusters, as well as ribosome binding sites at the 5’-ends.

### Construction of plasmids for flavonoids production

All constructed plasmids described below were verified by restriction enzymes digestions and also by sequencing of the cloned regions. *Streptomyces* producing clones were confirmed by PCR. Primers used amplify the first two common genes: 5’- GTGATCGAGCTGGACATGAA-3’ as the forward primer and 5’- GGCGTCCACGAGGTGC-3’ as the reverse primer.

### Construction of pKF

The plasmid pKF contains the ermE* promoter (P_ermE*_) and the 6 genes responsible for kaempferol biosynthesis. All synthetic gene cassettes were independently cloned in pUC57 and plasmids were named pLMF1 (pUC19 containing *TAL* gene), pLMF2 (*4CL*), pLMF3 (*CHS*), pLMF-FLS (*FLS1*), pLMF5 (*CHI*) and pLMF-N3DOX (*N3DOX*) (Table 1). Additionally, *TAL* gene was subcloned into vector pSL1180 as *Hind*III-*Bam*HI (pLMF7) to start with the cloning strategy. *4CL* gene (from pLMF2) was cloned into pLMF7 as *Pst*I-*Bam*HI gene cassette, generating pLMF8. Next step was subcloning FLS1 gene cassette from pLMF-FLS1 into pLMF3 as an *Eco*RI DNA fragment, giving rise to pKF11. The correct orientation of each DNA fragment was always confirmed by restriction enzymes digestions and sequencing. The two gene cassettes from pKF11 (*CHS* and *FLS1*) were subcloned together into pLMF8 as *Sac*I-*Bam*HI DNA band, in order to get the first 4 genes together in a plasmid (pKF14). Finally, *N3DOX* gene was subcloned into pLMF5 (opened *Eco*RV-*Bam*HI) as an *Eco*RI (blunt ended)-*Bam*HI gen cassette and the two genes together (*CHI* and *N3DOX*) were subcloned into pKF14 as *Xba*I-*Bam*HI resulting in the generation of pKF17, which contains the 6 genes required for kaempferol biosynthesis. As the expression host was *Streptomyces*, a further subcloning was required, and the *Bgl*II-*Bam*HI DNA fragment carrying the 6 genes was finally subcloned into pIAGO plasmid, a derivative of the bifunctional replicative vector pWHM3, which contains the *erm*E* promoter, giving rise to the final plasmid pKF.

**Table 1:** Plasmids and strains used in this study.

Plasmid	**Description**	**Source**
pIAGO	pWHM3 (replicative shuttle vector) carrying ermE* promoter	[45]
pSL1180	*E*. *coli* vector	[61]
pUC57	*E*. *coli* vector	Fermentas
pLMF1	pUC57 carrying *TAL*	This study
pLMF2	pUC57 carrying *4CL*	This study
pLMF3	pUC57 carrying *CHS*	This study
pLMF5	pUC57 carrying *CHI*	This study
pLMF-FLS	pUC57 carrying *FLS1*	This study
pLMF-N3DOX	pUC57 carrying *N3DOX*	This study
pLMF-F3H	pUC57 carrying *F3’H*	This study
pLMF-F35H	pUC57 carrying *F3’5’H*	This study
pLMF7	pSL1180 carrying *TAL*	This study
pLMF8	pSL1180 carrying *TAL* and *4CL*	This study
pKF11	pSL1180 carrying *CHS* and *FLS1*	This study
pKF14	pSL1180 carrying *TAL*, *4CL*, *CHS* and *FLS1*	This study
pKF16	pSL1180 carrying *CHI* and *N3DOX*	This study
pKF17	pSL1180 carrying *TAL*, *4CL*, *CHS*, *FLS1*, *CHI* and *N3DOX*	This study
pKF	pIAGO carrying *TAL*, *4CL*, *CHS*, *FLS1*, *CHI* and *N3DOX*	This study
pQR2	pSL1180 carrying *TAL*, *4CL*, *CHS*, *FLS1*, *CHI*, *N3DOX* and *F3’H*	This study
pQR	pIAGO carrying *TAL*, *4CL*, *CHS*, *FLS1*, *CHI*, *N3DOX* and *F3’H*	This study
pMYR2	pSL1180 carrying *TAL*, *4CL*, *CHS*, *FLS1*, *CHI*, *N3DOX* and *F3’5’H*	This study
pMYR	pIAGO carrying *TAL*, *4CL*, *CHS*, *FLS1*, *CHI*, *N3DOX* and *F3’5’H*	This study
pREC4	pSEVA98c1 containing *birA*, *accA2* and *accBE*	This study
**Strains**	**Description**	**Source**
*E*. *coli* TOP10	Strain used for routine sub-cloning and transformation in *S*. *albus*	Invitrogen
*E*. *coli* ET12567	Strain used for transformation in *S*. *coelicolor*	[43]
*Streptomyces coelicolor* M1154	Strain used to create the flavonols-producing mutants	[44]
*Streptomyces albus* J1074	Strain used to create the flavonols-producing mutants	[46]
*S*. *coelicolor*-pIAGO	*S*. *coelicolor* harboring pIAGO used as negative control	This study
*S*. *albus*-pIAGO	*S*. *albus* harboring pIAGO used as negative control	This study
*S*. *coelicolor*-pKF	*S*. *coelicolor* carrying pKF	This study
*S*. *coelicolor*-pQR	*S*. *coelicolor* carrying pQR	This study
*S*. *coelicolor*-pMYR	*S*. *coelicolor* carrying pMYR	This study
*S*. *albus*-pKF	*S*. *albus* carrying pKF	This study
*S*. *albus*-pQR	*S*. *albus* carrying pQR	This study
*S*. *albus*-pMYR	*S*. *albus* carrying pMYR	This study

### Construction of pQR

The plasmid pQR contains the ermE* promoter (P_ermE*_) and the 7 genes required for the biosynthesis of quercetin. To obtain this plasmid, it was required to add one more gene to the previous plasmid pKF17, synthetized to produce kaempferol. This gene, *F3’H*, was cloned into pKF17 as *Dra*I-*Bam*HI gene cassette giving rise to pQR2. The 7 genes contained in this plasmid were subcloned in the vector pIAGO to be further expressed in *Streptomyces*. The gene cassette was cloned as *Bgl*II-*Bam*HI DNA fragment to obtain the final plasmid pQR (Table 1).

### Construction of pMYR

The plasmid pMYR contains the ermE* promoter (P_ermE*_) and the 7 genes required for the biosynthesis of myricetin. To have the 7 genes together in the same vector, it was necessary to clone the *F3’5’H* gene into the previously constructed plasmid pKF17. The gene was cloned as *Dra*I-*Bam*HI gene cassette giving rise to pMYR2. The 7 genes contained in this plasmid were subcloned in the vector pIAGO to be further expressed in *Streptomyces*. The gene cassette was cloned as *Bgl*II-*Bam*HI DNA fragment to obtain the final plasmid pMYR (Table 1).

### Construction of pREC4 for malonyl-CoA metabolic engineering in *S*. *albus*

The plasmid pREC4 is a derivative of the *E*. *coli-Streptomyces* bifunctional vector pSEVA98c1 (colE1 and pIJ101 origins of replication for *E*. *coli* and *Streptomyces* respectively, both high copy number; apramycin resistance gene *aac(3)IV*). Vector pSEVA98c1 was digested with *Pac*I-*Sac*I in order to introduce the P_ermE*_ promoter for *Streptomyces*, giving rise to pREC3. Finally, pREC3 was digested with *Bam*HI-*Hind*III, in order to introduce the 5.1 kb DNA fragment containing the *S*. *coelicolor* chromosomal genes *birA* (biotin ligase, SCO4927 gene), *accA2* (alpha subunit of acetyl-CoA carboxylase, SCO4921 gene) and *accBE* (beta and epsilon subunits of acetyl-CoA carboxylase, SCO5535 and SCO5536 genes respectively) [49–51]. These plasmids were amplified by PCR from *S*. *coelicolor* chromosomal DNA, using the following primers, which contain sequences for restriction enzymes at their ends (marked in capital letters): birA-up (contains *Bgl*II recognition sequence): 5’-AGATCTcggcagtgcggtctttcccacac*-*3’, birA-rp (contains *Eco*RV-*Xba*I recognition sequence): 5’-GATATCaaaTCTAGAtcacgccaaccgcaggtgc-3’, accA2-up (contains *Eco*RV recognition sequence): 5’-GATATC taaactcggcttgtttcaagga-3’, accA2-rp (contains *Spe*I-*Xba*I recognition sequence): 5’-ACTAGTaaaTCTAGAgactgcttgatcagtccttga-3’, accBE-up (contains *Spe*I recognition sequence): 5’-ACTAGTgacggctcgcaatccttgctcg-3’, accBE-rp (contains *Xba*I recognition sequence): 5’-TCTAGAgttcgggtcagcgccagctg-3’.

### Extraction and analysis of flavonols

*S*. *albus* J1074 and *S*. *coelicolor* clones harboring pKF, pQR, pMYR and pIAGO (negative control), or pREC4 (for malonyl-CoA metabolic engineering) were cultivated (three replicas for each strain were extracted and quantified separately). In the case of liquid culture experiments, feeding experiments were carried out adding 0.3 mM final concentration (in the case of naringenin or kaempferol) or 0.01 mM (in the case of dihydrokaempferol) of these flavonoid precursors at 48 h-old cultures of the corresponding *S*. *albus* strain in 5 mL R5 medium, and incubating the cultures for another 72 h (liquid cultures) or 161 h (solid cultures). Flavonols extraction was carried out using three volumes of ethyl acetate with 0.01% of formic acid. This mixture was incubated for 1 h in orbital shaking at room temperature. After this incubation, the organic phase was concentrated by rotary evaporation and kept at -20°C for later use.

Dry extracts were dissolved in 200 μl of methanol:DMSO (1:1), filtrated (0.2 μm PVDF) and analyzed by liquid chromatography-electrospray ionization mass spectrometry (LC-ESI-MS/MS, Agilent technologies 1290 Infinity, Triple Quadrupole), which was carried out using a Zorbax Eclipse Plus C18 column (50 mm x 2.1 mm, 1.8 μm) in the negative ion mode. The analytes were eluted at a flow rate of 0.3 mL/min using a gradient of 0.1% (v/v) formic acid in water (A) and 0.1% (v/v) formic acid in acetonitrile (B) at 0–10% of B for 1 min, which was increased to 35% for 3 min and maintained at 35% for 1 min, then increased to 80% for 3 min and maintained at 80% for 2 min and finally decreased to 10% for 1 min. Flavonoids quantification was carried out in multiple reaction monitoring (MRM) mode in MS/MS. To accomplish this, the following ion sets were selected to detect the transitions of the parent ions to the product ions specific to the analytes: naringenin 272>119 Da and 272>151 Da; dihydrokaempferol 288>125 Da and 288>259 Da; dihydroquercetin 304>125 Da and 304>285 Da; kaempferol 286>93 Da and 286>117 Da; quercetin 302>151 Da and 302>179 Da; myricetin 318>151 Da and 318>179 Da; apigenin 270>117 Da and 270>150 Da; luteolin 286>131 Da and 286>151 Da. Authentic standards were purchased from Sigma Aldrich (naringenin and dihydrokaempferol), Calbiochem (dihydroquercetin) and Cayman Chemical Company (kaempferol, quercetin and myricetin).

## Results

### Heterologous expression of kaempferol

In microorganisms, the activity of four enzymes, TAL, 4CL, CHS and CHI, is required to produce naringenin, which is the main flavonols precursor (Fig 1). To obtain the flavonol kaempferol, the activity of other two enzymes is also needed. The first enzyme, N3DOX, hydroxylates naringenin in the position C3 to form the immediate kaempferol precursor, dihydrokaempferol. Later, FLS1 catalyzes de formation of a double bond between C2 and C3 to finally obtain kaempferol (Fig 1). In this work, all the six synthetic genes encoding for the enzymes required for the biosynthesis of kaempferol (with codon usage adapted to the transcription characteristics of *Streptomyces*) were cloned in a replicative high-copy number shuttle vector for *E*. *coli-Streptomyces*, under the control of P_ermE*_ (see Materials and Methods section). The final plasmid, pKF, was transformed and expressed in two different species of *Streptomyces*: *S*. *albus* and *S*. *coelicolor*.

**Fig 2:**
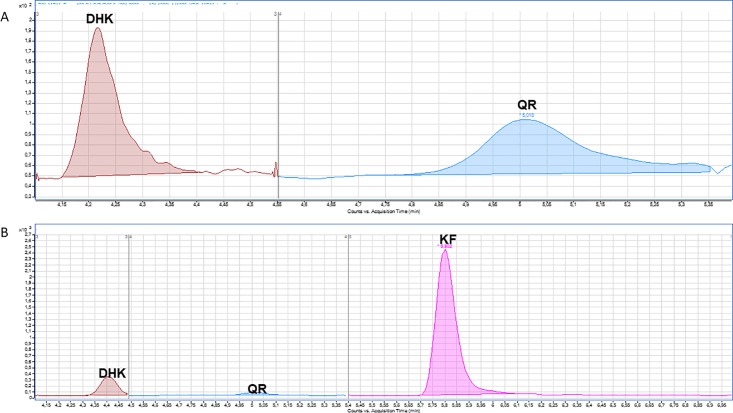
A: HPLC-MS chromatogram obtained after MRM analysis of the flavonols extracted from *S*. *coelicolor*-pKF. It shows the m/z peaks corresponding to dihydrokaempferol (DHK: <0.1 μM) and quercetin (QR: <0.1 μM). B: HPLC-MS chromatogram obtained after MRM analysis of the flavonols extracted from *S*. *albus*-pKF. It shows the m/z peaks corresponding to dihydrokaempferol (DHK: 0.039 μM), quercetin (QR: <0.2 μM) and kaempferol (KF: 0.212 μM).

Cultures of *S*. *albus*-pKF and *S*. *coelicolor*-pKF in R5A solid medium were analyzed by HPLC-MS chromatography in multiple reaction monitoring (MRM) in MS/MS mode, in order to identify and quantify the final product, kaempferol, as well as its intermediate precursors naringenin and dihydrokaempferol. The presence of the hydroxylated form of kaempferol, quercetin, was also analyzed. In *S*. *coelicolor*-pKF no kaempferol was detected. However, low production levels (below 0.1 μM) of the precursor, dihydrokaempferol, and the flavonol quercetin were observed (Fig 2A, Table 2).

**Table 2:** Concentrations of the different flavonoids detected in the both host bacteria for heterologous biosynthesis (mean values and standard error of the mean are indicated).

Plasmid	Host	Detected Flavonoids	Mean Concentration (μM) ± SEM	Mean Concentration (mg/L)
pKF	*S*. *coelicolor*	Dihydrokaempferol Kaempferol Quercetin	Below 0.1 - Below 0.1	Below 0.03 - Below 0.03
	*S*. *albus*	Dihydrokaempferol Kaempferol Quercetin	Below 0.1 0.212 ± 0.008 0.200 ± 0.016	Below 0.03 0.060 ± 0.002 0.060 ± 0.005
pQR	*S*. *coelicolor*	Dihydrokaempferol Quercetin Kaempferol	0.100 ± 0.010 0.100 ± 0.006 -	0.028 ± 0.003 0.030 ± 0.002 -
	*S*. *albus*	Dihydrokaempferol Quercetin Kaempferol	Below 0.1 0.340 ± 0.026 0.155 ± .0006	Below 0.03 0.102 ± 0.008 0.044 ± 0.002
pMYR	*S*. *coelicolor*	Dihydrokaempferol Quercetin Myricetin	Below 0.1 Below 0.1 Below 0.1	Below 0.03 Below 0.03 Below 0.03
	*S*. *albus*	Dihydrokaempferol Kaempferol Quercetin Myricetin Apigenin (with pREC4) Luteolin (with pREC4 plus naringenin feeding)	Below 0.1 Below 0.1 1.984 ± 0.014 0.146 ± 0.019 0.300 ± 0.021 Below 0.1	Below 0.03 Below 0.03 0.599 ± 0.004 0.046 ± 0.006 0.081 ± 0.006 Below 0.03

In *S*. *albus*-pKF, kaempferol was detected at 0.212 μM, but also dihydrokaempferol was detected below 0.1 μM and traces of quercetin (0.2 μM) (Fig 2B, Table 2). The presence of kaempferol, quercetin, myricetin and their precursors, naringenin and dihydrokaempferol was analyzed in parallel in the negative controls (*S*. *albus*-pIAGO and *S*. *coelicolor*-pIAGO). No flavonoids were detected in any case in these negative control strains.

### Heterologous expression of quercetin

Quercetin is a hydroxylated form of kaempferol. For its biosynthesis, it is only necessary the activity of one extra enzyme (F3’H), able to hydroxylate kaempferol in the position B3’. After checking that our *S*. *albus* host were able to produce kaempferol, the gene encoding for the F3’H was cloned into the plasmid directing the biosynthesis of kaempferol. The new plasmid, pQR, was transformed in both *S*. *albus* and *S*. *coelicolor* protoplasts. The cultures from positive recombinant strains (in R5A solid medium) were analyzed by HPLC-MS chromatography.

**Fig 3:**
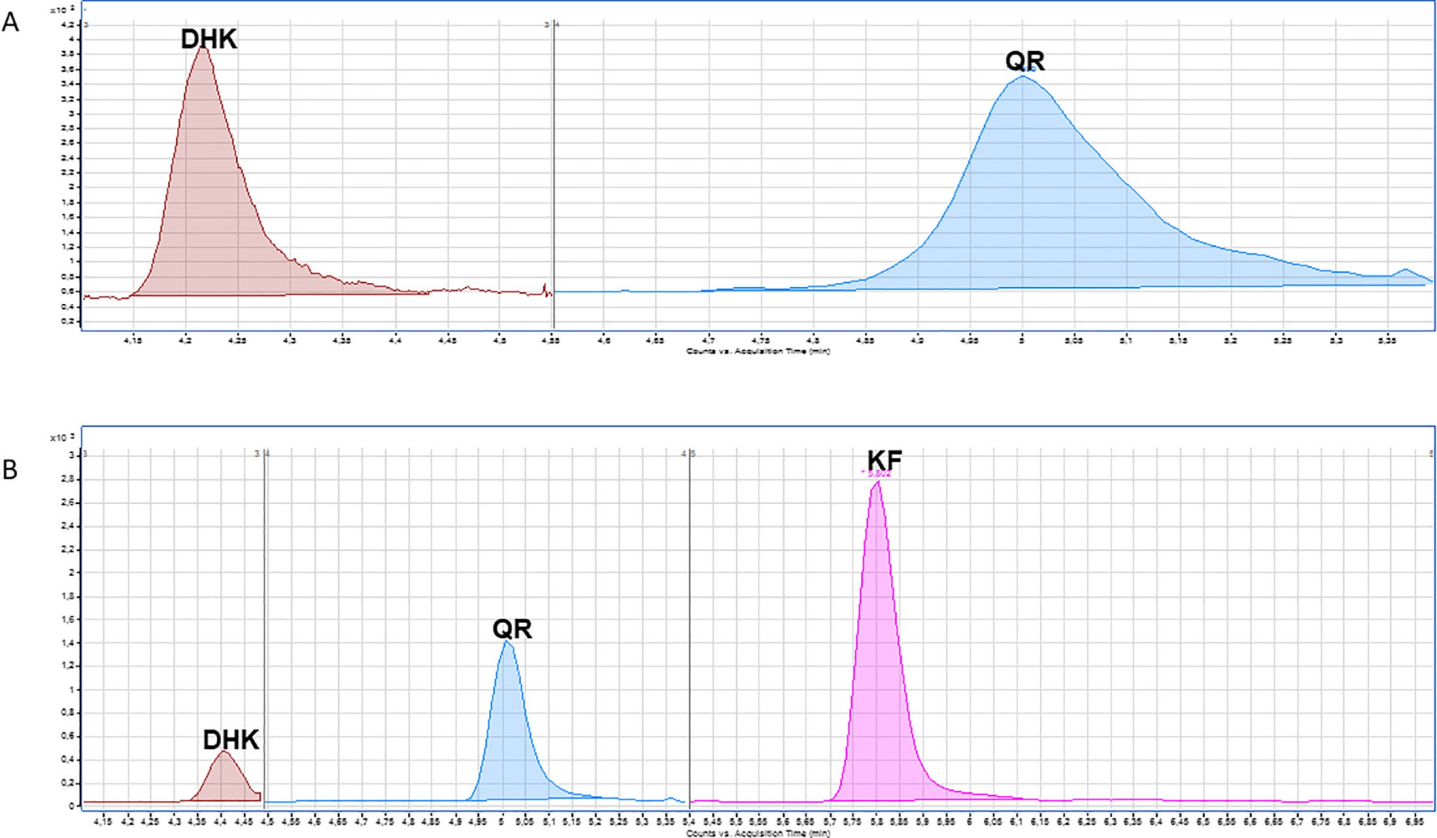
A: HPLC-MS chromatogram obtained after MRM analysis of the flavonols extracted from *S*. *coelicolor*-pQR. The m/z peaks correspond to dihydrokaempferol (DHK: <0.1 μM) and to quercetin (QR: <0.1 μM). B: HPLC-MS chromatogram obtained after MRM analysis of the flavonols extracted from *S*. *albus*-pQR. The peaks correspond to dihydrokaempferol (DHK: 0.047 μM), quercetin (QR: 0.340 μM) and kaempferol (KF: 0.155 μM).

In *S*. *coelicolor*-pQR, quercetin and one of the intermediate precursor, dihydrokaempferol were observed, although at levels below 0.1 μM (Fig 3A, Table 2).

Cultures of *S*. *albus*-pQR produced quercetin (0.34 μM), and its precursors kaempferol (0.155 μM) and dihydrokaempferol (below 0.1 μM) were also detected (Fig 3B, Table 2).

### Heterologous expression of myricetin

The flavonol myricetin, is also a hydroxylated form of kaempferol. In this case, there are two extra hydroxylations, while in quercetin there is only one. These two extra hydroxyl groups are in the positions B3’ and B5’, so the activity of a specific enzyme, F3’5’H, is required (Fig 1). The gene encoding this enzyme was added to the previous construction directing the biosynthesis of kaempferol and the new plasmid was further transformed in *S*. *coelicolor* and *S*. *albus*.

**Fig 4:**
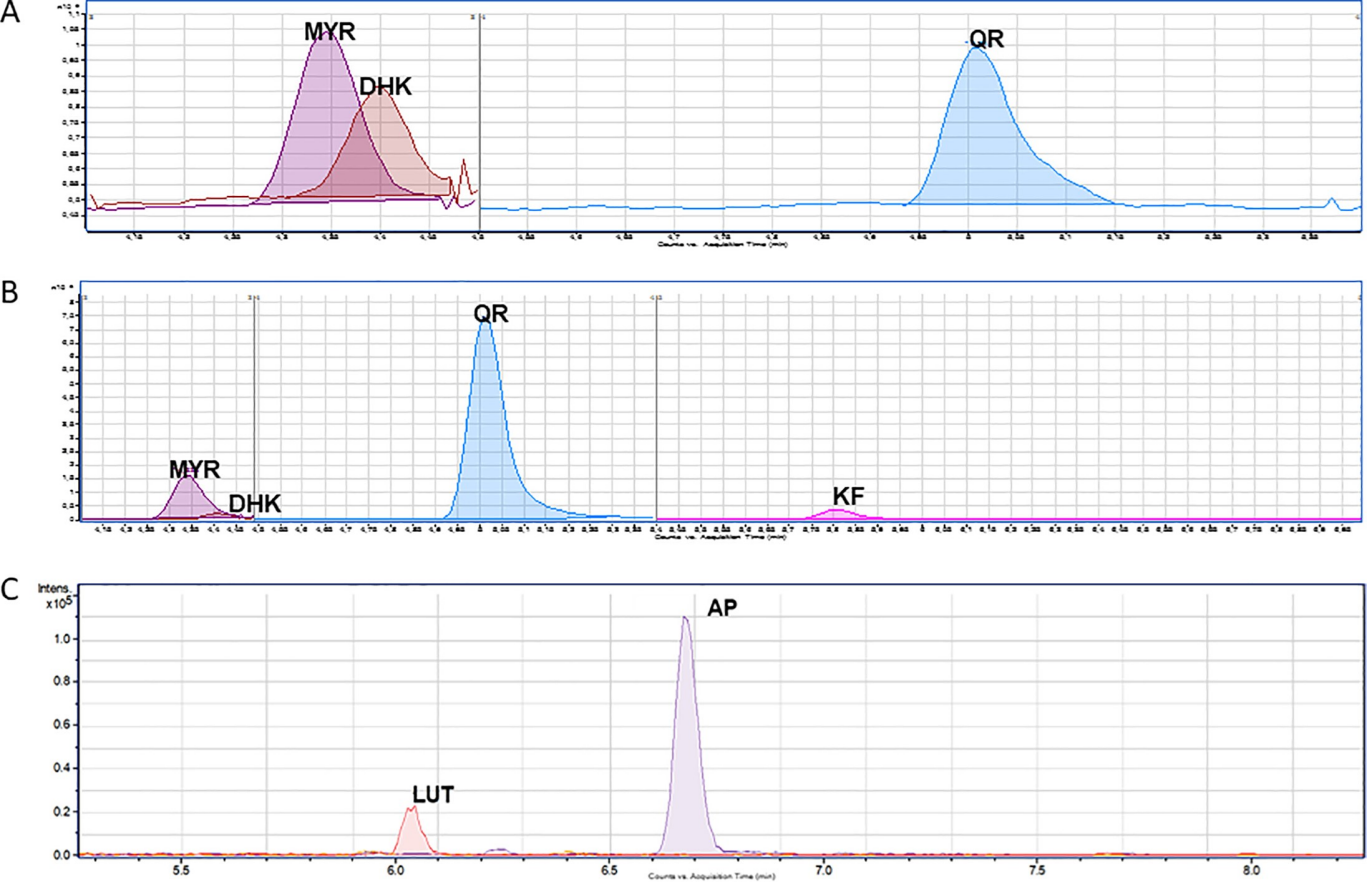
A: HPLC-MS chromatogram obtained after MRM analysis of the flavonols extracted from *S*. *coelicolor-*pMYR. The peaks correspond to myricetin (MYR: <0.1 μM), dihydrokaempferol (DHK: <0.1 μM) and quercetin (QR: <0.1 μM). B: chromatogram obtained after MRM analysis of the flavonols extracted from *S*. *albus-*pMYR. The peaks correspond to myricetin (MYR: 0.146 μM), dihydrokaempferol (DHK: 0.024 μM), quercetin (QR: 1.984 μM) and kaempferol (KF: 0.034 μM). C: chromatogram obtained from extracts of *S*. *albus-*pMYR-pREC4. The peaks correspond to apigenin (0.3 μM) and luteolin (below 0.1 μM).

In *S*. *coelicolor*-pMYR, myricetin was detected, but also the presence of dihydrokaempferol and quercetin was observed (Fig 4A). However, the levels of production were very low (under 0.1 μM was detected in MRM analysis).

Nevertheless, in *S*. *albus*-pMYR, higher levels of myricetin production were observed (0.146 μM). Other precursors, kaempferol and dihydrokaempferol are also produced by *S*. *albus*-pMYR in low concentrations (below 0.1 μM). However, the most abundant flavonol produced by this recombinant strain was quercetin (1.984 μM) (Fig 4B).

*S*. *albus*-pMYR was transformed also with plasmid pREC4, which contains all the genes coding for malonyl-CoA biosynthesis enzymes (BirA and acetyl-CoA carboxylase subunits). In this strain, however, no higher production levels of myricetin were detected in liquid cultures, but 0.3 μM of the shunt product apigenin, a derivative of naringenin (the precursor of dihydrokaempferol, kaempferol and myricetin) was detected. Apigenin was not detected in *S*. *albus*-pMYR- pSEVA98c1 (control strain for metabolic engineering experiments). The same strain, *S*. *albus*-pMYR-pREC4 was cultivated in R5 liquid medium as well, but including a feeding with naringenin. In this case, no further increase in apigenin production levels was detected, but interestingly, very low amounts of luteolin (a 3’-hydroxylated derivative of apigenin) were detected.

## Discussion

In this work, two different strains of *Streptomyces*, *S*. *albus* and *S*. *coelicolor*, have been able to biosynthesize *de novo* the flavonols kaempferol, quercetin (a 3’-hydroxylated kaempferol), myricetin (a double hydroxylated form of kaempferol) and the main precursor of the flavonols pathway, dihydrokaempferol.

Previous works reported the biosynthesis of kaempferol in *E*. *coli* [39,40], *Saccharomyces cerevisiae* [41] and *S*. *venezuelae* [42]. Two strategies were followed to produce kaempferol in *E*. *coli*. The first one consisted of adding L-Tyrosine (the first precursor of flavonoids in prokaryotes) to the culture medium, so the complete biosynthetic pathway was cloned. The levels of production obtained were up to 15 mg/L [40]. The group of Leonard *et al*. did not clone the gene encoding for the first enzyme of the biosynthetic pathway (tyrosine ammonia lyase, TAL), which means that feeding the cultures with precursors was needed. When *p*-coumaric acid (second metabolite of the biosynthetic pathway in microorganisms) was added, the yields of kaempferol reached 0.3 mg/L. The productions achieved were better (0.8 mg/L) when naringenin was used for feeding the culture [39].

Kaempferol was also produced in yeast in a similar way that it was synthetized in *E*. *coli*. The complete biosynthetic pathway was cloned in *S*. *cerevisiae* and three different precursors were added to the cultures: L-phenylalanine (first precursor of flavonoids in eukaryotes), *p*-coumaric acid and naringenin. The production levels achieved were 1.3 mg/L, 0.9 mg/L and 4.6 mg/L respectively [41].

Regarding the biosynthesis of kaempferol in *Streptomyces*, only one group succeeded to do it previously. However, the complete biosynthetic pathway was not cloned, only the genes encoding for the N3DOX and FLS1 were cloned in *S*. *venezuelae*. These enzymes are involved in the hydroxylation of naringenin to obtain dihydrokaempferol (N3DOX), and in the further formation of a double bond to form kaempferol (FLS1). So, it was required to supplement the cultures with naringenin precursor. The production levels reached 0.2 mg/L [42].

In this work we were able to produce kaempferol by cloning the complete biosynthetic pathway into *S*. *albus* without feeding the cultures with any precursor. However, the levels achieved were 0.212 μM. Also, smaller levels of its precursor dihydrokaempferol were observed, indicating that the enzyme FLS1 is not completely efficient. In *S*. *coelicolor*, no kaempferol was detected but small amounts of its precursor, dihydrokaempferol, and its hydroxylated derivative, quercetin, were detected. This may be due to the presence of an extra P450 hydroxylase naturally found in this strain that is able to use kaempferol as a substrate, thus, converting it into quercetin. This affirmation is supported by the fact that P450 systems are really well developed in *Streptomyces* genus, and P450 from this actinomycete has been described for regioselective hydroxylation of diverse flavonoids [52–54].

Regarding quercetin biosynthesis, it was achieved by the same groups in both *E*. *coli* and *S*. *cerevisisae* following the strategy employed to produce kaempferol [39,41]. In *E*. *coli*, the complete biosynthetic pathway, except for the first gene (encoding for TAL), was cloned, and cultures supplemented with either *p*-coumaric acid or naringenin. The production levels were 0.05 mg/L and 0.18 mg/L respectively [39].

Quercetin production in *S*. *cerevisiae* was higher. In this species, all the genes involved in quercetin biosynthesis were cloned. Like in the case of kaempferol production, the cultures were feed with L-Phe, *p*-coumaric acid and naringenin. Nonetheless, production levels were smaller. In the case of L-Phe feeding there were only traces of quercetin. When *p*-coumaric acid and naringenin were added, the production was up to 0.26 mg/L and 0.38 mg/L respectively [41].

In this paper, we describe the biosynthesis of quercetin in *Streptomyces* for the very first time. Moreover, it was *de novo* biosynthesis as no precursors were added to these cultures. However, only traces of quercetin were detected in the recombinant strain of *S*. *coelicolor*. In the case of *S*. *albus*, 0.1 mg/L of quercetin were observed, as well as relatively high amounts of kaempferol and dihydrokaempferol, indicating the incomplete efficiency of the P450 hydroxylases employed in this study. Although the amount of quercetin produced in this work is rather small, it is comparable to the one achieved in *E*. *coli* after being feed with naringenin [39], evidencing that our biosynthetic system is more effective.

As far as the biosynthesis of myricetin in microorganisms is concerned, this flavonol was only produced by the group of Leonard *et al*., in *E*. *coli*, and all the genes required to produce myricetin were introduced in *E*. *coli* and then, the cultures were supplemented with naringenin and eriodictyol. Despite this feeding, production levels only reached 0.01 mg/L [39].

In our case, we demonstrate the feasibility of *de novo* myricetin biosynthesis in both *S*. *albus* and *S*. *coelicolor*. Moreover, production levels (0.146 μM) in *S*. *albus* were better than those achieved in *E*. *coli* even after feeding experiments. In *S*. *albus*, not only myricetin was detected by HPLC-MS, but also high levels of its precursor, quercetin (1.984 μM). This elevated amount of precursor in comparison to the final compound may be due to a 3’-hydroxylation activity of the enzyme F3’5’H in the C3’. In fact, it is known that *P*. *hybrida* F3’5’H performs both 3’- and 3’,5’-hydroxylation reactions and can use flavonols as well as dihydroflavonols and flavanones as a substrate [39,55]. Taking into account these two considerations, the high yield of quercetin could be due to a 3’-hydroxylation of the intermediate dihydrokaempferol generating dihydroquercetin as a product which could be easily converted in quercetin by the FLS1 enzyme expressed in pMYR plasmid. Moreover, more quercetin can be produced from kaempferol due to, once again, a 3’-hydroxylation of the F3’5’H. For all these reasons, only a little quantity of kaempferol is available to be converted in myricetin by F3’5’H. Finally, it should be pointed out that *P*. *hybrida* F3’5’H has a broad substrate specificity towards dihydrokaempferol, kaempferol and quercetin but competition as well as inhibition may occur when more than one substrate is present, leading to a lower myricetin yield and quercetin accumulation [55].

In our experiments with *S*. *coelicolor* there are only traces of these compounds. These results, together with the obtained ones for the biosynthesis of kaempferol and quercetin, reveal that *S*. *albus* is a better host for flavonols biosynthesis than *S*. *coelicolor*. Also, low production levels in *S*. *albus* could be improved by metabolic engineering of the strain, facilitating the incorporation of malonyl-CoA to the flavonols biosynthetic pathway, as it is a limiting factor in flavonoid production [56]. Other authors confirmed that this strategy is useful to increase flavonoids yields [37,40,57,58]. However, the experiments with *S*. *albus*-pMYR-pREC4, where plasmid pREC4 was used to try to further increase intracellular malonyl-CoA precursor levels, and therefore to generate higher myricetin titers, were unsuccessful. In these experiments, instead of higher myricetin levels, due to the expected higher malonyl-CoA intracellular levels, a deviation from naringenin precursor towards apigenin (a shunt product in this study) was observed. A possible explanation for this result is that the enzyme N3DOX, in charge of converting naringenin intermediate towards hihydrokaempferol, shows a 79.18% identity with the enzyme FNS, which usually converts naringenin towards apigenin [59,60]. This means that genes present in pMYR can explain the generation of apigenin in *S*. *albus*-pMYR-pREC4 as a shunt product from naringenin. Once apigenin is present at those levels in *S*. *albus*-pMYR-pREC4, further feeding here with exogenous naringenin facilitates apigenin production and the low levels of the apigenin 3’-hydroxy derivative luteolin observed. This 3’-hydroxylation can be easily explained by the presence of F3’5’H hydroxylase in pMYR.

Alternatively, production levels could be also improved by facilitating hydroxylation steps carried out by F3’H and F3’5’H enzymes. These two enzymes need reducing power provided by a single redox partner (cytochrome P_450_ enzyme) whose presence is critical to show their optimal or maximal activities [39,54], and this reductase is not included in our plasmid constructions. In fact, there is a big hurdle to combine specific P450s with the right redox partners. Nevertheless, the partial activity of the P450 hydroxylases used in this study could be explained by a recognition of soluble endogenous redox partners since P450 systems are specially well developed in *Streptomyces* genus [52,54]. Further modifications of these two enzymes involving their transformation in a soluble chimera protein that fuses the P450s with suitable P450 reductases are being carried out.

## Conclusions

Using a combinatorial biosynthesis approach and reconstituted plant flavonol pathways in the bacteria *S*. *albus*, this work describes the heterologous biosynthesis of the important nutraceuticals myricetin and quercetin by the first time in actinomycetes, according to public literature. Also, kaempferol biosynthesis has been achieved in this bacterium for the first time without feeding with precursors. These experiments open the way to heterologous production in actinomycetes of other flavonols and flavonoids.
